# Life science database cross search: A single window system for dispersed biological databases

**DOI:** 10.6026/97320630015883

**Published:** 2019-12-31

**Authors:** Jun-ichi Onami, Hideki Hatanaka, Shoko Kawamoto, Toshihisa Takagi

**Affiliations:** 1 Japan Science and Technology Agency, National Bioscience Database Center, Japan; 2 Research Organization of Information and Systems, Database Center for Life Science, Joint-Support Center for Data Science Research; 3 Research Organization of Information and Systems, Department of Informatics, National Institute of Genetics, Japan; 4 Toyama University of International Studies, Japan

## Abstract

A comprehensive search system for the bioscience databases is in progress. We constructed a search service, Life science database cross search system (https://biosciencedbc.jp/dbsearch/index. php?lang=en) by integrating numerous biomedical databases using database crawling algorithms. The described system integrates 600 databases containing over 90 million entries indexed for biomedical research and development.

## Background

The cross-search service of bioscience databases is still developing. Because data are considerably dispersed across various organizations and networks, finding required information immediately is difficult. Additionally, conducting comprehensive searches in large bioscience databases using general web search engine such as Google is difficult [1]. Some search-related infra structure such as BioCaddie [2] and World Wide Science [3], which are dedicated to research, have been constructed. Because of the deep web problem [4], these search results are not comprehensive and efficient. In this project, we collected all the data from selected bioscience web databases that contained entries in the deep web and developed a web search engine that could search the compiled and comprehensive bioscience database.

## Methodology

**Figure 1:**
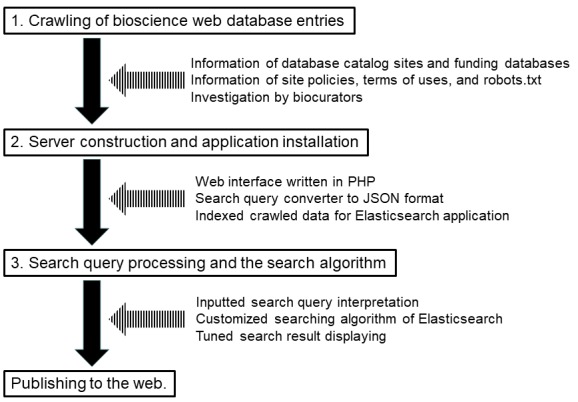
The flowchart of search system construction and the flow of information and technology

This web service is constructed in three steps as shown in (Figure 1) and the details are described below.

## Web data crawling:

URLs of bioscience web database entries were collected from database catalog sites and funding databases. We checked their site policies, terms of uses, and robots.txt and evaluated the pros and cons of crawling web data by well-known algorithm [5]. Then, biocurators investigated each entry in the database and distinguished the data containing text that was suitable for text search. Additionally, they checked the variation range of URLs. For instance, some URLs comprised sequential numbers in a predetermined number of digits and padding zero or well-known identifiers in the bioscience field such as PDB ID or Uniprot ID. Database crawling scripts were programmed with a compiled URL list. If a database entry contained some useful metadata such as "species name," "gene name," or "date of creating the database entry" the script parsed each metadata for storage. These metadata were classified into bioscience categories, so that the category of each database could be distinguished.

## Server construction and application installation:

A web server and a search server were constructed.

## [A] Web server:

The input interface containing the search box was written in PHP. If a user inputted some words into the text box, related words were suggested via an internal dictionary. The search query was parsed, converted into JSON format, and properly processed (as described in Section 3) to be inputted to the Elasticsearch application [6].

## [B] Search server:

The search server parses the outputted JSON data from the Elastic search and displays the search results with a parsed title and snippet fields for visibility. The web interface was published with the page of the target database list and the help page written in English and Japanese. Elasticsearch version 1.7 was installed into the search servers. The crawled data were analyzed by a bigram tokenizer and indexed. The cluster structure that comprised multiple servers accomplished load balancing and data redundancy. The search server receives a search query request from the web server and inputs it into the search process of Elastic search.

## Search query processing and the search algorithm:

The search query that is inputted to the search box is subjected to Boolean interpretation, word distinction, and stop word removal in the web server. In this process, if the search query contains words that are often used in the field of bioinformatics (e.g., gene and database), the ranking score of the related database category is boosted. The search results are displayed from Elasticsearch in the order of a decreasing score.

## Availability:

Users can retrieve comprehensive information of a matched text by inputting a keyword query into the search box from Life science database cross search system [7]. Detailed information is available via the link of the search result snippet. The index is automatically updated using the batch script when the updates of the original site have been detected or the running annual updates have been recognized on the basis of RSS or update history. This extensive service would be helpful as an academic search infrastructure for researchers who need to access comprehensive entries in the bioscience database and for users from the intellectual property department. This type of domain-specific search infrastructure has been expensive to construct, and the legal decision of crawling each database has been difficult to make. However, search infrastructure has recently become ubiquitous for global commercial search engines and has become prevalent in ordinary society. Search engine optimization technologies such as "robots.txt" have been well understood. The importance of crawling is well known because data-driven analytic studies are becoming common. Japanese copyright law has been revised and the legitimacy of data crawling has been clarified. In this situation, Life science database cross search is the only service that provides high quality search capabilities to access large quantities of bioscience data. The expectation that the bioscience database must continuously distill to this infrastructure is reasonable.

## Conclusion

We describe the development and use of a comprehensive search system integrating 600 databases containing over 90 million entries indexed for biomedical research and development using database crawling algorithms.
